# Beyond diagnosis: is there a role for radiomics in prostate cancer management?

**DOI:** 10.1186/s41747-023-00321-4

**Published:** 2023-03-13

**Authors:** Arnaldo Stanzione, Andrea Ponsiglione, Francesco Alessandrino, Giorgio Brembilla, Massimo Imbriaco

**Affiliations:** 1 Department of Advanced Biomedical Sciences, University of Naples Federico II, Naples, Italy; 2 Department of Radiology, Jackson Memorial Hospital, University of Miami, Miami, FL USA; 3 Department of Radiology, IRCCS San Raffaele Scientific Institute, Vita-Salute San Raffaele University, Milan, Italy

**Keywords:** Artificial intelligence, Clinical decision-making, Prostatic neoplasms, Radiomics, Reproducibility of results

## Abstract

The role of imaging in pretreatment staging and management of prostate cancer (PCa) is constantly evolving. In the last decade, there has been an ever-growing interest in radiomics as an image analysis approach able to extract objective quantitative features that are missed by human eye. However, most of PCa radiomics studies have been focused on cancer detection and characterisation. With this narrative review we aimed to provide a synopsis of the recently proposed potential applications of radiomics for PCa with a management-based approach, focusing on primary treatments with curative intent and active surveillance as well as highlighting on recurrent disease after primary treatment. Current evidence is encouraging, with radiomics and artificial intelligence appearing as feasible tools to aid physicians in planning PCa management. However, the lack of external independent datasets for validation and prospectively designed studies casts a shadow on the reliability and generalisability of radiomics models, delaying their translation into clinical practice.

**Key points**

• Artificial intelligence solutions have been proposed to streamline prostate cancer radiotherapy planning.

• Radiomics models could improve risk assessment for radical prostatectomy patient selection.

• Delta-radiomics appears promising for the management of patients under active surveillance.

• Radiomics might outperform current nomograms for prostate cancer recurrence risk assessment.

• Reproducibility of results, methodological and ethical issues must still be faced before clinical implementation.

## Background

Prostate cancer (PCa) is the second most common cancer and the fifth cause of cancer-related death in men worldwide [1]. PCa shows a highly heterogeneous clinical behaviour, ranging from indolent disease [2] to treatment-resistant lethal disease [3]. A wide range of management options are available, ranging from deferred treatment, such as active surveillance (AS), to primary treatment with curative intent, including radical prostatectomy (RP) and radiotherapy (RT) and systemic therapy (hormonotherapy and chemotherapy) [4]. In this context, an accurate patient selection is key to deliver the most appropriate management in terms of oncologic outcomes and quality of life.

Despite multiparametric magnetic resonance imaging (mpMRI) and prostate-specific membrane antigen (PSMA)-ligand positron emission tomography/computed tomography (PET/CT) have revolutionised the diagnostic pathway of PCa, their role in preoperative staging, treatment planning, and in the PCa recurrence setting is less defined [5, 6]. Currently, therapeutic and prognostic recommendations widely rely on risk-stratification tools based on clinical parameters such as clinical stage, prostate-specific antigen (PSA), and Gleason score (GS) [7–9]. Nonetheless, there is mounting evidence to suggest that imaging can improve accuracy of clinical-based prognostic models [10, 11]. Still, there are some limitations of imaging to be addressed in order to exploit its full potential in this setting, such as the inherent subjectivity, variability of image interpretation, and lack of reliable quantitative parameters.

To address these issues, radiomics has been proposed as an image analysis approach that allows a high-throughput extraction of objective quantitative features (morphological, statistical, and textural) that are missed by human eye [12, 13]. Radiomics features have the potential to better describe tumour phenotype and its heterogeneity, providing relevant diagnostic and prognostic information to better inform clinical decision-making [14].

While the bulk of radiomics research has been mainly focused on PCa detection and characterisation [15], there are many potential implications of radiomic features in management planning for both primary and recurrent PCa, which could lead to a more personalised treatment approach (Table 1). For example, features of tumour aggressiveness could inform on local and nodal staging, overcoming the limited sensitivity of preoperative imaging, with direct impacts on the choice of the surgical technique (*e.g.*, nerve-sparing, pelvic lymph node dissection). Radiomics and machine learning (ML)-based solutions could assist radiation oncologist in their daily practice, from treatment planning to toxicity prediction. Also, a better characterisation of tumour aggressiveness may help in defining which patients may benefit from adjuvant therapies, to reduce post-treatment recurrence [14, 16, 17]. Finally, radiomics features may help identifying metastatic lesions that are more likely to respond to systemic therapies, and to quantify the effectiveness of a specific treatment.

**Table 1:** Main settings in which radiomics and artificial intelligence could play a role for prostate cancer patient management, based on currently available evidence, with brief definitions of management strategies as discussed in this review

	What is it	Which patients	Possible issues	Potential applications of Radiomics/AI
**Radiotherapy (RT)**	Radiation-based radical treatment (external beam radiotherapy, brachytherapy)	Locally confined primary disease; adjuvant treatment in patients undergoing RP; men with BCR after RP	Urinary and gastrointestinal toxicity, disease recurrence	Target delineation, treatment planning and dose optimisation, assessment of response
**Radical prostatectomy (RP)**	Surgical removal of the prostate and the seminal vesicles (+/- pelvic lymph nodes)	Locally confined, intermediate-risk disease; selected cases of low-risk or high-risk and locally advanced disease	Urinary incontinence, erectile dysfunction, disease recurrence	Pre-operative risk stratification, loco-regional staging, surgical planning
**Active surveillance (AS)**	Close monitoring of organ confined PCa (PSA, clinical examination, MRI, biopsy)	Men with life expectancy > 10 y and low-risk disease; selected cases of intermediate-risk disease	Inappropriately deferred curative radical treatment, disease progression	Risk stratification for patient enrolment, evaluation of disease progression
**Biochemical recurrence (BCR)**	Rising PSA after primary treatments with curative intent (RP and RT)	Patients who underwent primary treatments with curative intent	Suboptimal predictive models, low sensitivity of imaging for recurrence detection	BCR prediction, recurrence detection

*AI* Artificial intelligence, *PCa* Prostate Cancer, *PSA* Prostate specific antigen

**Fig. 1:**
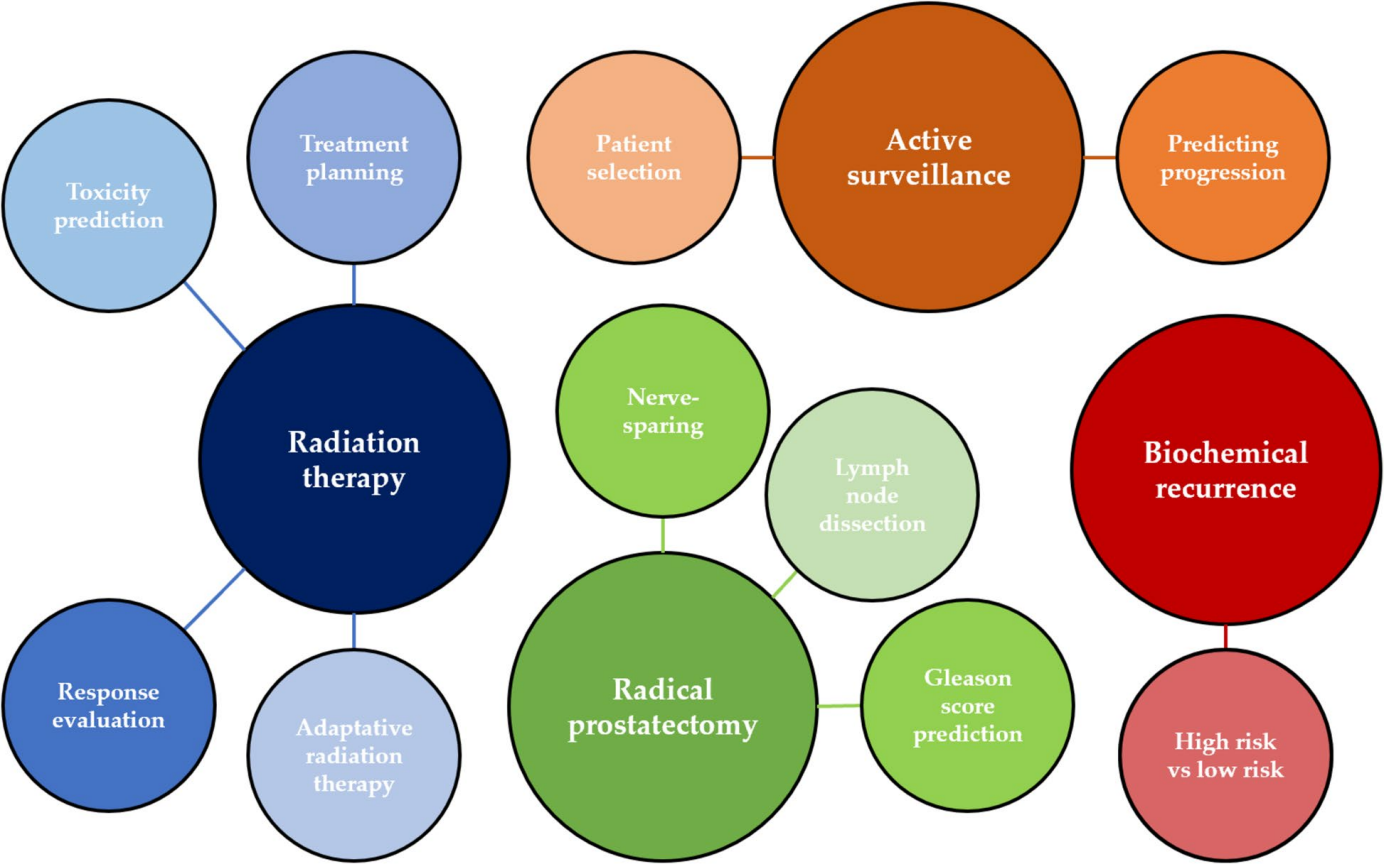
Graphic representation of radiomics and artificial intelligence main applications in the setting of prostate cancer management

In this narrative review, we present the current knowledge on the potential application of radiomics for PCa treatment planning, with a management-based approach and a highlight on recurrence of disease after treatment, while also covering some artificial intelligence (AI)-based imaging tools when relevant (Fig. 1).

### Radiomics and radiotherapy

RT has a consolidated role in cancer treatment, and it can be estimated that around half of all oncologic patients will receive RT either alone or in combination with other treatments [18]. In this setting, PCa patients represent no exception. Diagnostic imaging has historically played a major role in all aspects of RT, from identification of eligible patients and therapy planning to the assessment of treatment response, and the evolution of RT is closely tied to the advancements in diagnostic imaging [19]. For example, it is foreseeable that MRI will become more relevant for RT in the near future, due to its advantages for target definition [20] as well as for dose adaptation and personalisation, with MRI-linear accelerator (MRI-LINAC) systems being commercially available [21]. Similarly, radiomics is expected to transform the way RT is currently conceived, bringing an added value to many tasks in the RT workflow which could benefit from novel imaging biomarkers ML decision support models [22]. Indeed, radiomics could assist radiation oncologist in the transition toward personalised medicine, allowing to tailor treatment on individual patients based on their specific needs, thus possibly improving outcome while reducing toxicity [23].

### Treatment planning

Radiomics pipeline and RT workflow share a common time-consuming task that is prone to error and low reproducibility due to inter-operator variability: image segmentation. Usually, radiomics requires segmentation of the primary tumour lesion, frequently corresponding to the target of RT. However, when planning treatment, the radiation oncologist also needs to consider the critical normal structures located in proximity of the actual target that might be damaged by RT, defined as organs at risk (OAR), further increasing the workload. Automated segmentation tools have been gaining interest in the field of prostate MRI. Different deep learning (DL) algorithms for whole-gland as well as for lesion segmentation have been proposed, with more than promising results and some commercial solutions already available (*e.g.*, DynaCAD Prostate®, Philips, Amsterdam, The Netherlands, Quantib® Prostate, Quantib B.V. Rotterdam, The Netherlands) [24–26].

In particular, the imaging paradigm for RT planning in PCa patients is shifting from computed tomography to MRI, and a number of solutions based on AI have been proposed to automatise the MRI segmentation process with the aim of reducing treatment planning time, decreasing the workload for radiation oncologists and possibly promoting more consistent outcomes [27, 28]. In 2019, Elguindi et al. [29] employed transfer learning to train, test, and then externally validate a DL algorithm (DeepLabV3+, https://hasty.ai/docs/mp-wiki/model-architectures/deeplabv3) using contours manually annotated by an experienced radiation oncologist. The DeepLabV3+ was able to automatically segment the prostate and seminal vesicles (volumetric dice similarity coefficient 0.83 ± 0.06) as well as five OAR including bladder, rectum, urethra, penile bulb, rectum/rectal spacer. Similarly, DL has been proposed as a feasible tool to improve accuracy and consistency of MRI target and OAR segmentations for PCa RT planning in clinical trials, automatically flagging delineations needing corrections thus reducing the workload for radiation oncologists performing quality assurance (with sensitivity and specificity for target volumes needing major corrections of 0.73 and 0.86, respectively) [30].

With specific regard to OAR, Savenije et al. [31] trained two DL algorithms to segment bladder, rectum, and femurs and compared their performance to that of an atlas-based software. They found that one of the algorithms (DeepMedic, https://deepmedic.org/)) was faster and more accurate compared to the benchmark, with segmentations requiring fewer manual corrections. Of note, after training and testing they were able to successfully translate DeepMedic into clinical practice and did not observe a decrease in the automated segmentation tool’s performance compared to the experimental setting.

A very recent publication postulated that such strategies will likely disrupt daily practice in the near future, reporting a first-in-human experience of completely autonomous unsupervised treatment planning approach to deliver MRI-guided RT to a PCa patient [32]. In this experience, both OAR and target volume were automatically contoured by a DL tool and a baseline treatment plan was autonomously generated using particle swarm optimisation. No human interaction was required up to treatment plan optimisation and plan approval by the radiation oncologist. The time from simulation to treatment was inferior to 6 h and the automate treatment plan fulfilled most of the dosimetry criteria adopted for quality assurance check.

However, it is important to further deepen our understanding of these tools, which should not be superficially deemed as perfect or super-human. Indeed, a recent study revealed that multiple DL algorithms have the highest segmentation variability in those anatomical regions (*e.g.*, junctions between prostate and bladder or the external urinary sphincter) in which interobserver variability is the highest for radiation oncologists [33]. This finding is somewhat expected when considering that human drawn contours are usually employed to train the automatic segmentation algorithms.

### Adaptative RT and assessment of response

Delivering the most appropriate dose based on individual tumour features is among the goals of personalised RT. Surely, heterogeneity is a hallmark of cancer, with focal variations in angiogenesis, hypoxia, and thus metabolism which contribute to determine tumour aggressiveness and treatment response [34]. Radiomics holds the promise to characterise tumour heterogeneity and paired to ML could be used effectively to identify and automatically segment target areas corresponding to the MRI index lesion for dose boosting during RT, as proposed by Shiradkar et al. [35] with the Rad-TRaP framework.

The idea of refining and adjusting treatment plan to account for new clinical and imaging information that become available over time is commonly referred to as *adaptive RT*, and has been historically limited by the lack of readily available data on tumour biological changes [36]. MRI-LINAC and radiomics might offer a solution to overcome this limitation. Indeed, beyond the clear advantages of better motion management and precise tumour localisation, allowing for safe dose escalation, the use of MRI-LINAC implies the creation of novel imaging datasets daily enriched with new scans. These datasets represent the ideal starting point for radiomics studies aimed at the prognostic assessment of cancer patients [37].

**Fig. 2:**
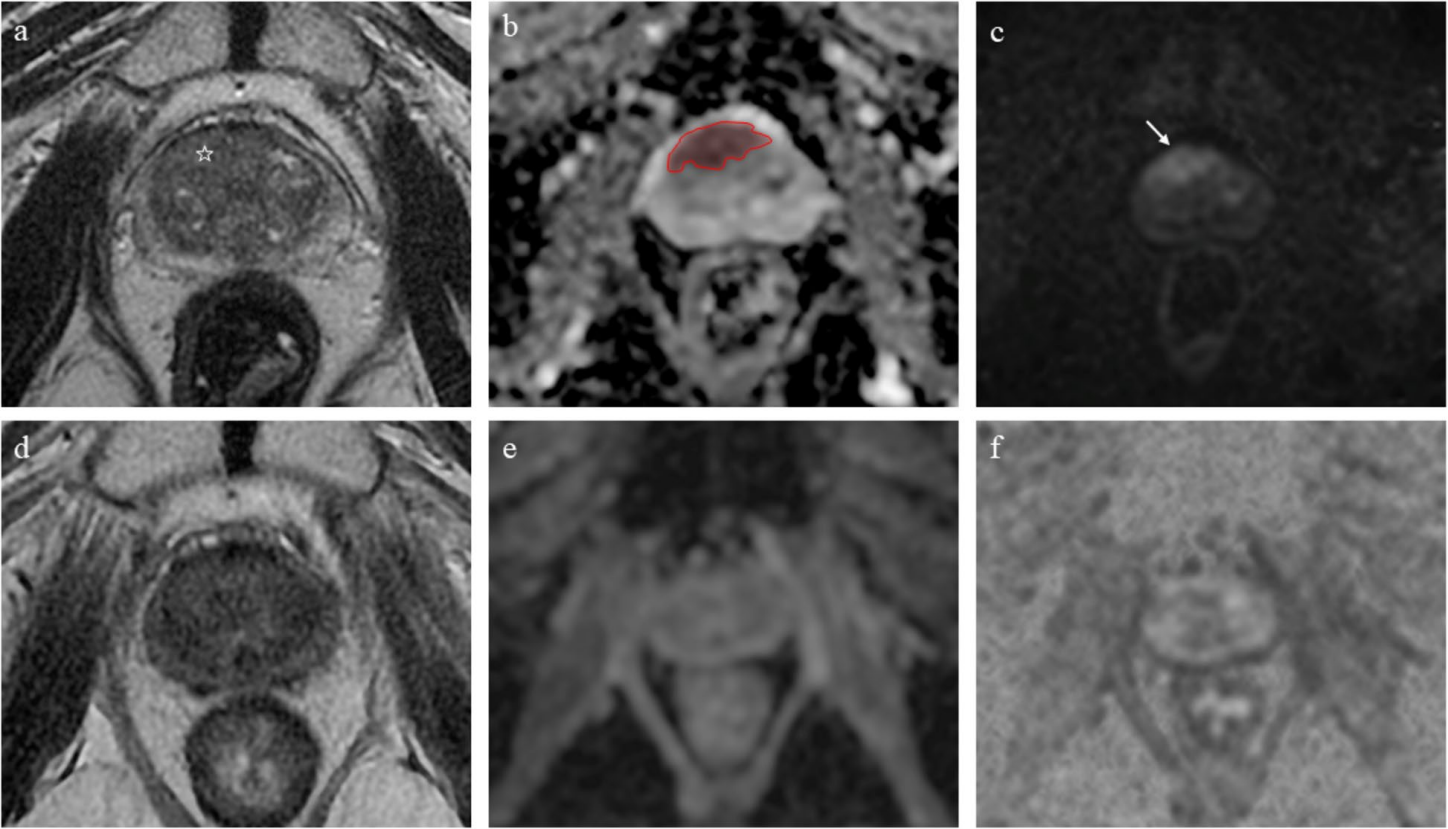
Sixty-nine-years old patient treated with external beam radiotherapy after MRI targeted biopsy revealed 3+4 Gleason score prostate cancer. Images from pre-biopsy (**a**−**c**) as well as post-treatment (**d**−**f**) MRI scans are presented. The index lesion (PI-RADS 5) can be appreciated on the T2-weighted images (**a**, lesion epicentre marked with a white star) as a homogeneous, moderately hypointense area with obscured margins in the right anterior transition zone. Corresponding marked and focal hypointensity on ADC map (**b**, red region of interest) and hyperintensity on high *b* value DWI (**c**, white arrow) are present. On the corresponding post-treatment sequences (**d**−**f**), no abnormalities can be detected, suggesting a good treatment response. *DWI* Diffusion-weighted imaging, *MRI* Magnetic resonance imaging, *PI-RADS* Prostate Imaging-Reporting and Data System

In particular, *delta-radiomics* (the study of changes in radiomics features over time) feasibility experiments with multiple time points could be performed. Using two time points only (before and after treatment), a recent study on 33 PCa patients found that delta-radiomics is outperformed by radiomics approaches based on pre- or post-treatment images alone in the prediction of response to RT (Fig. 2) [38]. However, this study might have been limited by the relatively small sample size as well as the number of available time points and future experiments are needed to investigate whether radiomics of multiple MRIgRT images can provide and added prognostic value for PCa management, as recently found for pancreatic cancer [39]. Notwithstanding, researchers venturing in this field should be aware of the challenges to be faced. There are differences between MRI scans acquired for diagnostic purposes and those from MRI-LINAC scanners. For example, diffusion weighted imaging (DWI) acquisition is technically challenging for MRI-LINAC scanners [40] and only a minority of radiomics features are stable and robust on MRI-guided RT feature selection [41]. Test-retest studies and great care in feature selection will be required to ensure high methodological standards are met.

### Toxicity

To minimise the side effects of RT, radiomics and ML approaches have been proposed to predict OAR toxicity [42]. Regarding PCa RT, only preliminary investigations have been carried out to explore the role of MRI radiomics. In particular, a pilot delta-radiomics study on 30 patients treated with RT for PCa found that radiomic features extracted from femoral head volumes exhibit significant differences between pre- and post-RT MRI scans [43]. While the authors argue that these variations could be related to RT-induced biological changes, the lack of feature robustness for temporal variation and the absence of meaningful clinical correlates (*i.e.*, fractures) do not allow to make strong claims regarding the value of radiomics for predicting RT bone toxicity.

Another delta-radiomics study was similarly designed for the evaluation of radiation-induced changes in the bladder wall using T2-weighted imaging (T2WI) [44]. However, beyond changes in radiomics features values between pre- and post-RT MRI, the correlation between radiomics features and radiation dose as well as radiation induced urinary toxicity (*i.e.*, cystitis) was evaluated and an overall good correlation was found in both cases. The same research group also performed a pilot study on radiomics for rectal RT toxicity prediction in PCa patients and found that a combined model employing both T2WI and DWI features extracted from pre-RT MRI had a good prediction power, with an area under the curve (AUC) of 0.81, thus possibly representing a field worthy of further investigation for the pretreatment prediction of rectal toxicity [45]. Finally, MRI radiomics has been reported as a promising strategy to identify patients at higher risk of developing urethral strictures as late adverse effect of high dose brachytherapy for PCa treatment in a case-control study [46]. Specifically, on pre-treatment MRI, statistically significant differences emerged between the stricture cases and controls for radiomics features like contrast and homogeneity while no correlation with urethral dosimetry was found.

Quantitative computational features can be extracted from all types of medical images, and since volumetric maps of RT dose levels distributions are indeed images a complementary approach to radiomics defined as *dosiomics* has been proposed [47]. In 2018, Rossi et al. [48] tried to improve the prediction of genitourinary and gastrointestinal toxicity of PCa RT. With a cohort of 351 patients, they found that adding dosiomics to non-treatment related parameters (*e.g.*, age, previous treatment) significantly increased the accuracy of rectal bleeding and faecal incontinence prediction compared to using non-treatment-related parameters alone (AUC of 0.58 *versus* 0.73 and 0.63 *versus* 0.73 respectively). Similarly, adding dosiomics to non-treatment-related parameters increased the prediction accuracy for urinary incontinence (AUC of 0.68 *versus* 0.73), although statistical significance was not reached in this case. Taken together, these findings suggest that dosiomics should not be neglect and deserves consideration, possibly in the context of multi-omics models [49].

### Radiomics and radical prostatectomy

Treatment choices in PCa patients are guided by risk stratification, which is based on PSA levels, GS and clinical stage [4]. RP represents the main option alongside RT for PCa primary treatment with curative intent. An overview of the main RT and RP studies discussed in this review is presented in Table 2. With specific regard to RP, it is recommended as a valid option for active treatment with curative intent for patients at low-intermediate risk. Conversely, for high-risk patients or locally advanced disease, RP should be considered in selected cases and in the context of a multimodal therapy.

**Table 2:** Main studies on radiomics/AI imaging applications for primary treatments with curative intent in the setting of prostate cancer management (one example for each main potential application is proposed)

First author [reference number]	Publication year	Country	Treatment	Aim	Design	Sample size	Imaging modality	Main outcome	Potential impact
Künzel [32]	2021	Germany	RT	Autonomous treatment planning	Prospective, single-centre	1	MRI (AI)	Feasibility of autonomous treatment planning for adaptive MRIgRT proven	Reduction of time to treatment and RO workload. Potential first step toward real-time RT
Shiradkar [35]	2016	USA	RT	Targeted focal treatment planning	Retrospective, multicentre	23	MRI (radiomics)	Radiomics framework showed reduction in dosage to OARs and boosted dose to index lesions	Decision support framework for RO to elaborate more effective and targeted treatments
Rossi [48]	2018	The Netherlands	RT	Toxicity prediction	Retrospective, multicentre	351	Dose distributions (dosiomics)	Dosiomics showed an added value for the prediction of RT toxicity, statistically significant for GI toxicity compared to NCTP	More reliable prediction of adverse events might aid RO in treatment planning
Abdollahi [38]	2019	Iran	RT	Prediction of treatment response	Retrospective, single-centre	33	MRI (radiomics)	Pre-treatment MRI radiomics might identify non-responders to IMRT (AUC 0.78)	Different treatment options could be preferred for potential non-responders
Solari [52]	2021	Germany	RP	Prediction of GS upgrade from Bx	Retrospective, single-centre	101	[^68^Ga] Ga-PSMA-11 PET/MRI (radiomics)	Combined PET+ADC radiomics model outperformed Bx in the prediction of GS at RP (accuracy of 82.5% *versus* 72.4%)	More reliable patient risk stratification could be achieved to guide management
Cuocolo [59]	2021	Italy	RP	Detection of EPE	Retrospective, multi-centre	193	MRI (radiomics)	Radiomics model for EPE detection showed good generalizability in a multicentre setting and might aid radiologists in PCa staging	Low EPE probability at pre-treatment MRI might help select suitable candidates for nerve-sparing surgery
Zheng [67]	2022	USA	RP	Evaluation of lymph node involvement	Retrospective, single-centre	244	MRI (radiomics)	A combined model (clinical and radiomics features) outperformed pre-existing nomograms for the prediction of lymph node status (AUC 0.915 *versus* 0.724)	Reducing the number of unnecessary ePLND identifying patients with low probability of lymph node involvement

*AI* Artificial intelligence, *AUC* Area under the receiver operating characteristic curve, *Bx* Biopsy, *EPE* Extraprostatic extension of disease, *ePLND* Extended pelvic lymph node dissection, *GI* Gastrointestinal, *GS* Gleason score, *IMRT* Intensity-modulated radiotherapy, *MRIgRT* MRI-guided radiotherapy, *NTCP* Logistic normal tissue complication probability, *OARs* Organs at risk, *RO* Radiation oncologist, *RP* Radical prostatectomy, *RT* Radiotherapy, *TP* Treatment planning

However, the current risk stratification model is not exempt from limitations. Among these, GS at biopsy has been reported to be prone to undergrading compared to the final score assigned at RP, possibly leading to high-risk patients being selected for RP [50]. In this context, recent studies suggest that radiomics could potentially represent a complementary tool to biopsy allowing for a more accurate preoperative GS assessment [51, 52]. Using multiparametric 3-T MRI radiomics and multivariate logistic regression analysis on 166 PCa patients treated with RP, Zhang et al. [51] build (*n* = 116) and validated (*n* = 50) a predictive model which showed a good performance (AUC 0.87) in the prediction of biopsy GS upgrade at RP. When adding the radiomics signature and clinical parameters into a nomogram, the predictive performance further improved (AUC 0.91). While these findings are encouraging, it should be considered that MRI-targeted biopsies were not performed in this study, which could have led to an overestimation of biopsy GS downgrading in this study. Furthermore, the relatively low number of high GS (> 7) in the study cohort did not allow to perform a subgroup analysis, which might have confirmed the added value of radiomics.

Another very recent work compared the performance of different whole-gland radiomics models based on PSMA PET/MRI to that of biopsy for the prediction of GS at RP in 101 retrospectively enrolled PCa patients [52]. Among the different single-modality and combined models trained in this IBSI-Image Biomarker Standardization Initiative compliant study, the PET plus apparent diffusion coefficient (ADC) model outperformed biopsy (AUC 82.5% *versus* 72.4%) in the prediction of GS at RP. While integrated PET/MRI scanners are not widespread, the reported findings support the hypothesis that multimodal radiomics might support urologists in the risk stratification of PCa patients. Radiomics might also be able to predict the occurrence of bone metastases on pretreatment MRI scans, as found in a population of PCa patients under watchful waiting, outperforming GS alone as a predictor [53]. The predicted risk of distant tumour spread could be helpful identify patients eligible to more aggressive treatment strategies.

### Nerve-sparing surgery

**Fig. 3:**
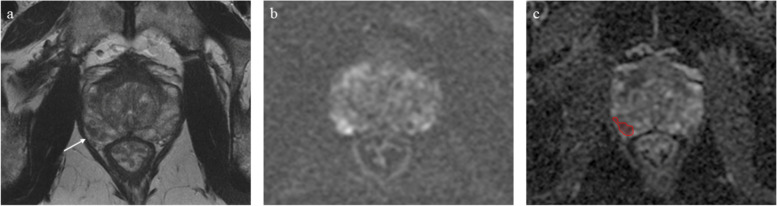
Prebiopsy MRI scan of a 58-year-old patient (PSA value of 6.12 ng/mL at the time of imaging) showing a PI-RADS 4 lesion in the posterior-lateral peripheral zone (right lobe) appearing hypointense on T2-weighted images (**a**) and exhibiting markedly restricted diffusion (**b**, high *b* value DWI; **c**, ADC map with red region of interest). Target biopsy confirmed the presence of prostatic adenocarcinoma (Gleason score 3 + 4). While a moderate capsule-tumour contact length can be appreciated on the T2-weighted images (**a**, white arrow), no bulging nor definitive signs of extracapsular extension are present and the radiologist staged the disease as locally confined. Based on the MRI report and considering the young age, the patient underwent nerve-sparing radical prostatectomy. Unfortunately, the pathology report on the surgical specimen revealed the presence of extracapsular disease extension and upgraded the Gleason score to 4 + 4. *ADC* Apparent diffusion coefficient, *DWI* Diffusion-weighted imaging, *MRI* Magnetic resonance imaging, *PI-RADS* Prostate Imaging-Reporting and Data System, *PSA* Prostate-specific antigen

Bilateral preservation of the neurovascular bundles can lead to better urinary and sexual function outcomes without compromising cancer control [54, 55]. However, current guidelines recommend against performing nerve-sparing surgery when extracapsular extension is suspected [4]. MRI provides important and useful information regarding extracapsular extension of PCa, with various signs being used in different scoring system with good diagnostic performances [56, 57], but the inter-reader agreement is rather low and the accuracy is linked to the experience of radiologists [58]. Radiomics could offer feasible solutions to overcome these limitations and thus help identify patients not eligible for nerve sparing surgery (Fig. 3) [59–61].

In a recent study, a support vector machine model was trained to detect the presence of extraprostatic extension disease using radiomic features extracted from index lesions volumes as identified on the T2WI and ADC map of preoperative MRI scans [59]. The overall accuracy ranged from 83% (train set) to 74–79% (test set), not statistically different from that of an experienced radiologist. Considering the multicentre and multiscanner setting, these findings indicate a good model generalisability and a possible benefit to support less experienced readers might be hypothesised. In line with these results, subsequent studies from different research groups confirmed that MRI radiomics analysis might deliver valuable information regarding extracapsular extension of PCa, which can be successfully paired with clinical parameters to obtain more holistic models with even greater predictive performances [60, 61].

### Lymph node dissection

At present, it is still unclear how beneficial lymph node removal may be on PCa outcomes and while the procedure can be justified for the information it provides regarding cancer spread, it is associated with complications [62]. Several nomograms can be used to assess the risk of nodal involvement in PCa, in order to select candidates for lymph node dissection and its extent, but most of them were developed in the pre-MRI era [63]. The conventional evaluation of lymph node on imaging strongly relies on size and morphology as criteria for involvement, suffering from poor sensitivity [64].

Radiomics has been assessed as a potential strategy to improve the role of imaging [65–67]. Whole gland ADC radiomics obtained from automatically annotated volumes of interests was not statistically different in terms of accuracy when compared to two of these nomograms, although the overall accuracy is still lower than desirable (AUC 0.73) [65]. With a different approach, Zheng and colleagues [67] integrated radiomic features, extracted from the index lesions on T2WI and ADC map, and clinical features (*e.g.*, PSA, biopsy results) to build a support vector machine model. In this case, the integrated model achieved an AUC of 0.915, significantly higher compared to that of clinical nomograms whose highest AUC was 0.724. The results of a previous study from a different research group combining T2WI and ADC index lesion radiomics with clinical variables had already suggested the feasibility of this approach for lymph node status prediction and its superiority compared to conventional nomograms, although with differences in terms of model (*e.g.*, a neural network was used) [66]. It should be noted that all these studies were only internally validated. Until external validation is performed, the degree of generalizability remains uncertain and represents a possible major issue.

### Radiomics and active surveillance

AS has been proved to be a viable alternative to radical treatments such as RP or RT for low risk PCa, with similar oncological outcomes [68, 69]. AS protocol requires strict patients monitoring over time to recognise any potential risk reclassification that would need deferred radical intervention, still with curative purpose [69]. Annual biopsies identify whether patients on AS show upgrading or upstaging of PCa. However, due to the possible complications of the procedure as well as the risk of not correctly targeting the lesion of interest, in the last decade there has been an ever-growing interest in non-invasive diagnostic tools, such as MRI, enabling re-evaluation of the risk of PCa progression [70, 71]. Indeed, mpMRI has been included in several AS protocols [72, 73]. Moreover, the UK NICE (National Institute for Care and Clinical Excellence) currently recommends mpMRI either for baseline evaluation of AS candidates or for the assessment of clinical as well as PSA modifications during surveillance protocol [74].

Quantitative imaging techniques may provide objective measures of the underlying biological changes occurring over the course of natural history of PCa [75–78]. An overview of the main studies in the setting of AS discussed in this review is presented in Table 3. In their retrospective study, Xie et al. [75] assessed a combination of texture features and ML-based analysis of ADC maps for the prediction of grade group (GG) upgrading in GS ≤ 6 PCa (GG1) and GS 3 + 4 PCa (GG2) from biopsy to RP in 59 patients eligible for AS. Among the four supervised ML methods employed, the nearest neighbor algorithm, including six texture features (variance, skewness, kurtosis, 90% percentile, variance of absolute gradient, and S difference variance), showed the best diagnostic performance (AUC 0.71) in the test cohort for non-invasively prediction of PCa GG upgrading.

**Table 3:** Main studies on radiomics applications in the setting of active surveillance of prostate cancer (one example for each main potential application is proposed)

First author [reference number]	Publication year	Country	Aim	Design	Sample size	Imaging modality	Main outcome	Potential impact
Xie [75]	2021	China	Patient selection	Retrospective, single-centre	59	MRI (radiomics)	A combination of texture features and ML-based analysis of ADC maps could predict PCa GG upgrading from Bx to RP	ML models may help clinicians select appropriate therapeutic strategy
Sushentsev [77]	2021	UK	Predicting progression	Retrospective, single-centre	64	MRI (radiomics)	PRECISE and delta-radiomics models achieved comparably good performance for predicting PCa progression in AS patients (AUC 78–84.4%)	To provide a quantitative assessment of MRI-guided follow-up AS patients, less dependent on reader experience

*AS* Active surveillance, *Bx* Biopsy, *GG* Grade group, *ML* Machine Learning, *PCa* Prostate Cancer, *PRECISE* Prostate Cancer Radiological Estimation of Change in Sequential Evaluation, *RP* Radical prostatectomy

Moreover, Sushentsev et al. [77] compared the performance of the PRECISE scoring system against MRI-derived delta-radiomics models for predicting histopathological proven PCa progression in 64 patients on AS protocol with a median follow-up of 46 months. In detail, three delta-radiomics models, including 34 T2WI- and 53 ADC-derived texture features, were developed using the parenclitic networks, LASSO (least absolute shrinkage and selection operator regression), and random forests ML methods. The Authors showed that PRECISE scoring (AUC 84.4%) and delta-radiomics models (AUC 78.0−81.5%) yielded comparably good performance for predicting PCa progression in AS patients.

In a more recent investigation, Algohary et al. [78] evaluated the performance of MRI-based radiomics features (including Gabor, first-order statistics, and grey-level co-occurrence-based texture features) in identifying the presence of csPCa in 56 patients on AS regimen who had previously undergone prebiopsy 3-T biparametric MRI (T2WI plus DWI). In detail, the authors performed two experiments. Experiment 1 aimed to identify radiomics features able to discriminate patients with biopsy proven clinically significant PCa, while experiment 2 evaluated the ability of the selected radiomics features to identify the presence or absence of clinically significant disease in the more challenging cases with discordance between PI-RADS assessment and biopsy findings (groups 3 and 4). Out of the three ML models used, quadratic discriminant analysis yielded the best results, showing an overall accuracy improvement of 80%, while of 60% for groups 3 (MRI negative and biopsy positive) and 4 (MRI positiveand biopsy negative) when compared to PI-RADS v2.0 alone.

### Radiomics and biochemical recurrence

**Fig. 4:**
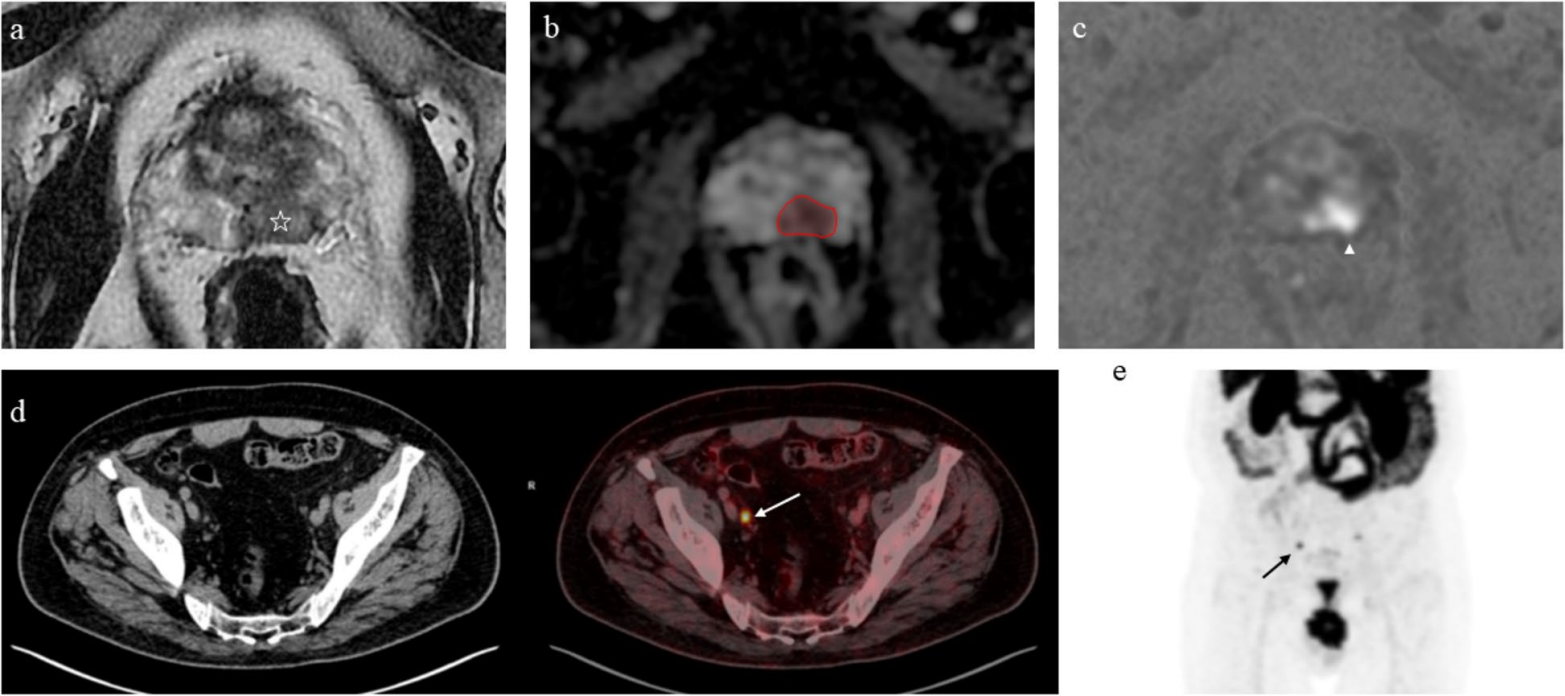
Seventy-two-year-old patient who underwent radical prostatectomy for prostate cancer (at pathology, Gleason score 3 + 4). Staging MRI shows a large peripheral zone (left posteromedial) lesion with intermediate signal on T2-weighted images (**a**, lesion epicentre marked with white star), focal and marked hypointensity on the ADC map (**b**, red region of interest) and corresponding hyperintensity on high *b* value DWI (**c**, white arrowhead). Three years after treatment, rise of PSA value determines biochemical recurrence. [^68^Ga]Ga-PSMA-11 PET/CT (**d** and **e**) shows a small but concerning right external iliac lymph node. *ADC* Apparent diffusion coefficient, *DWI* Diffusion-weighted imaging, *MRI* Magnetic resonance imaging, *PET/TC* Positron emission tomography/computed tomography, *PMSA* Prostate-specific membrane antigen, *PSA* Prostate-specific antigen

Both RP and RT are considered definite treatments for localised PCa [79]. However, about 27−53% of patients show biochemical recurrence (BCR) after those primary therapies (Fig. 4) [80, 81]. BCR definition varies according to the main curative interventions. After RP, the threshold is represented by PSA > 0.4 ng/mL that is rising [82]. Instead, the Radiation Therapy Oncology Group-American Society for Therapeutic Radiology and Oncology Phoenix consensus conference set the definition of BCR after primary RT as any PSA increase > 2 ng/mL higher than the PSA nadir, regardless of the nadir value [83].

An early identification of subjects at high risk for BCR may allow a better management of the disease, discriminating patients that would benefit the most of adjuvant RT from those who could avoid unneeded complementary treatments and the associated side effects [84]. To date, there are still a few studies aimed at radiomics prediction of BCR, mostly focused on MRI features either after RP [85, 86] or RT. [87] An overview of the main investigations in the setting of BCR discussed in this review is presented in Table 4.

**Table 4:** Main studies on radiomics applications in the setting of biochemical recurrence of prostate cancer after primary treatments with curative intent (one example for each imaging modality and/or treatment is proposed)

First author [reference number]	Publication year	Country	Treatment	Aim	Design	Sample size	Imaging modality	Main outcome	Potential impact
Li [88]	2021	USA	RP	BCRFS prediction	Retrospective, multicentre	198	MRI (radiomics)	A prognostic nomogram, incorporating pre-operative bpMRI radiomics features and clinicopathologic parameters outperformed CAPRAs score for BCRFS prediction (C-index 0.79 *versus* 0.68)	Identifying patients at low risk of BCR who might defer additional therapy
Fernandes [91]	2018	The Netherlands	RT	5-year BCR prediction	Retrospective, single-centre	120	MRI (radiomics)	LR model using whole-prostate MRI features (AUC 0.63) outperformed both clinical and combined models (AUC 0.51 and 0.56 respectively)	To develop individualised treatment strategies, stratifying patients at risk of BCR
Kang [93]	2020	USA	RP	BCR prediction	Retrospective, multicentre	28	F-18 fluciclovine PET/CT (radiomics)	The model combining Haralick texture features with patients’ clinical parameters improved BCR prediction compared to the models including only clinical data and imaging features (AUC 0.94, 0.71, and 0.92 respectively)	Developing a computational methodology to be used as an adjunct tool to improve and standardise the interpretation of F-18 fluciclovine PET/CT in the identification of BCR
Papp [92]	2020	Austria	RP	BCR prediction	Prospective, single-centre	52	[^68^Ga] Ga-PSMA-11 PET/MRI (radiomics)	Supervised predictive model for BCR, including PET/MRI features and clinical data outperformed the standard routine analysis based on PSA, biopsy GS, and TNM staging (diagnostic accuracy 0.89 *versus* 0.69)	Identifying patients at risk of BCR who might benefit additional therapy

*AUC* Area under the receiver operating characteristic curve, *BCRFS* Biochemical recurrence-free survival, *bpMRI* Biparametric MRI, *GS* Gleason score, *CAPRAs* Post-surgery cancer of the prostate risk assessment, *LR* Logistic regression, *PSA* Prostate specific antigen, *RP* Radical prostatectomy, *RT* Radiotherapy

In their retrospective multicentre study, Li et al. [88] developed and validated a prognostic nomogram, incorporating radiomics features extracted from biparametric MRI with preoperative clinicopathologic parameters, for predicting BCR free survival after RP in 198 patients with PCa. In detail, the nomogram was built with five radiomics features, including two T2WI Laws features, T2WI intensity range and Haralick information measure as well as ADC Laws feature. Their prognostic nomogram outperformed the Cancer of the Prostate Risk Assessment score (CAPRA) (C-index 0.79 and 0.68, respectively) and achieved comparable performance as the post-surgery CAPRA score (C-index 0.75) in a head-to-head comparison for BCR free survival prediction.

Another study group from France developed and externally validated an MRI ADC map-derived radiomics model to predict BCR and BCR free survival after RP [89]. Interestingly, while the radiomics model resulted to be predictive of BCR (accuracy of 0.76%), the clinical model failed to validate the external cohort (accuracy of 0.56%). Surprisingly, the radiomics-clinical model did not outperform the radiomics alone model, with an accuracy of 0.67%.

Moreover, Yan et al. [85] developed and externally validated a DL-based radiomics signature, including MRI features (first-order, shape, texture, wavelet, and Laplacian of Gaussian Filter) extracted from T2WI, to predict BCR of 485 patients underwent RP in three different Institutions. The radiomics model achieved a C-index of 0.802 in both primary and validating cohorts and outperformed the post-surgery CAPRA score (0.677), National Comprehensive Cancer Network model (0.586), and Gleason grade group system (0.583).

Promising evidence from MRI-derived radiomics features have been provided even in the setting of predicting BCR after RT. [87, 90, 91] Fernandes et al. [91] investigated the potential of whole-prostate imaging features extracted from the original and filtered T2W MR images for 5-year BCR prediction after RT of 120 patients with localised PCa. The logistic regression model built using whole-prostate imaging features (AUC 0.63) outperformed both the clinical and combined models (AUC 0.51 and 0.56, respectively).

Efforts for predicting BCR after primary therapies have been made with hybrid imaging, including PET/CT and PET/MRI [92–94]. Kang et al. [93] investigated the role of a computational methodology using Haralick texture analysis as an adjunct tool to improve and standardise the interpretation of F-18 fluciclovine PET/CT in identifying BCR of 28 patients with PCa underwent RP with or without subsequent salvage therapies. Of note, the Authors showed that the model combining Haralick texture features computed with patients’ clinical parameters improved the chances of accurately detecting BCR (AUC 0.94) compared to the models including only clinical data and imaging features (AUC 0.71 and 0.92, respectively).

Finally, in a recent investigation, part of a single-centre pilot to a randomised prospective trial, Papp et al. [92] investigated the diagnostic performance of [^68^Ga]Ga-PSMA-11 PET/MRI *in vivo* models for predicting low-*versus*-high lesion risk as well as BCR of 52 patients with PCa underwent RP with a ML approach. Their supervised predictive model for BCR selected seven radiomic features (coefficient of variation, grey level co-occurrence matrix information correlation type 1, standardised uptake value max, grey level co-occurrence matrix joint entropy, standardised uptake value mean, and high grey zone emphasis from the [^68^Ga]Ga-PSMA-11 images, interquartile range from ADC images) and clinical data. This model outperformed the standard routine analysis based on PSA, biopsy GS, and TNM staging (diagnostic accuracy of 0.89 and 0.69, respectively).

## Conclusions

Radiomics is still in its infancy, but it is foreseeable that AI and radiomics solutions will become a key component of radiologists’ everyday work in the future. Overall, the majority of research efforts have been focused on PCa detection [95, 96]. However, the advantages of radiomics might be even greater in the setting of treatment management, possibly compensating for the limitations of currently available strategies.

It could be speculated that studies on PCa detection are more common because it might be easier to obtain good quality datasets to work on (*e.g.*, more data is needed for studies on treatment response, such as follow-up data). It is also possible that less encouraging results have been found for more complex classification tasks and these are not emerging due to publication bias, a well-known issue in the field of radiomics [97].

The limitations of current evidence should be taken into account, and overly optimistic claims should be avoided. Indeed, each step in the radiomics pipeline hidden methodological pitfalls to be aware of [98]. Above all, the lack of external independent datasets for validation and of prospectively designed studies cast a shadow on the reliability and generalisability of radiomics models, hindering their translation into clinical practice. High image quality is a key factor for reliable prostate MRI conventional interpretation but is not easy to ensure [99]. Similarly, high-quality datasets are necessary to minimise the risk of a garbage in garbage out effect for radiomics and AI models [100]. Additionally, a substantial heterogeneity among radiomics studies was also found, for example in terms of methodology and transparency, but the scientific community is working to promote standardisation in imaging AI and radiomics research, with checklists and guidelines to help design, assess, and interpret radiomics papers [101–103].

Finally, medical-legal guidance to address the liability for clinical practice use of radiomics has not been yet provided by official regulatory offices but could aid physicians in gaining confidence with these tools and encourage their safe use [104].

In conclusion, this review highlighted excellent future prospects for a role of radiomics in powering decision support tools to aid physicians in the management and treatment planning of PCa patients. However, great efforts are advocated to confirm these encouraging premises and eventually produce the high-level evidence required to turn these exciting perspectives into medical practice realities.

## Data Availability

The data are available from the corresponding author on reasonable request.

## Abbreviations

ADC: Apparent diffusion coefficient
AS: Active surveillance
AUC: Area under the curve
BCR: Biochemical recurrence
CAPRA: Cancer of the prostate risk assessment
DL: Deep learning
DWI: Diffusion-weighted imaging
GG: Grade group
GS: Gleason score
ML: Machine learning
mpMRI: Multiparametric MRI
MRI: Magnetic resonance imaging
MRI-LINAC: MRI-linear accelerator
OAR: Organ at risk
PCa: Prostate cancer
PET/CT: Positron emission tomography/computed tomography
PI-RADS: Prostate Imaging Reporting and Data System
PSA: Prostate-specific antigen
PSMA: Prostate-specific membrane antigen
RP: Radical prostatectomy
RT: Radiotherapy
T2WI: T2-weighted imaging
